# Health service delivery and political trust in Nigeria

**DOI:** 10.1016/j.ssmph.2019.100382

**Published:** 2019-03-28

**Authors:** Adanna Chukwuma, Thomas J. Bossert, Kevin Croke

**Affiliations:** a Health, Nutrition, and Population Global Practice, World Bank Group, Washington, DC, 20433, USA; b Department of Global Health and Population, Harvard T. H. Chan School of Public Health, Boston, MA, 02115, USA

**Keywords:** Trust, Social services, Africa, Nigeria, Health, Politics

## Abstract

Do improvements in health service delivery affect trust in political leaders in Africa? Citizens expect their government to provide social services. Intuitively, improvements in service delivery should lead to higher levels of trust in and support for political leaders. However, in contexts where inadequate services are the norm, and where political support is linked to ethnic or religious affiliation, there may be weak linkages between improvements in service delivery and changes in trust in political leaders. To examine this question empirically, we take advantage of a national intervention that improved health service delivery in 500 primary health care facilities in Nigeria, to estimate the impact of residence within 10 km of one or more of the intervention facilities on trust in the president, local councils, the ruling party, and opposition parties. Using difference-in-difference models, we show that proximity to the intervention led to increases in trust in the president and the ruling party. By contrast, we find no evidence of increased trust in the local council or opposition parties. Our study also examines the role of ethnicity and religious affiliation in mediating the observed increases in trust in the president. While there is a large literature suggesting that both the targeting of interventions, and the response of citizens to interventions is often mediated by ethnic, geographic or religious identity, by contrast, we find no evidence that the intervention was targeted at the president's ethnic group, zone, or state of origin. Moreover, there is suggestive evidence that the intervention increased trust in the president more among those who did not share these markers of identity with the president. This highlights the possibility that broad-based efforts to improve health services can increase trust in political leaders even in settings where political attitudes are often thought to be mediated by group identity.

## Highlights

• The intervention was not targeted at the president's ethnicity or state of origin.
• Service improvements led to increases in trust in the president and ruling party.
• Trust increased among respondents outside the president's ethnicity or state of origin.

## 1 Background

How do improvements in health service delivery affect political trust and does group identity influence this relationship in Africa? Citizens expect their government to deliver social services. Intuitively, improvements in health service delivery should increase support for the government, including political trust. Much of the literature exploring the relationship between trust and health services has focused on trust among actors within a health system, which is conceptually distinct from trust in political leaders (Calnan & Rowe, 2006; Gilson, 2006; Rowe & Calnan, 2006). As health systems are part of the social fabric of countries, interpersonal relationships within them reflect the wider set of societal values and norms, and greater trust in the health system can contribute to building social trust in society more broadly (Gilson, 2003). However, few papers have examined the relationship between service delivery and trust in the politicians who hold power over the entire health system. We take advantage of a national maternal and child health intervention in Nigeria to examine this question. This research is closely linked with the growing literature that estimates the returns in terms of votes or approval levels to politicians following investments in health and other social services in developing countries, with mixed findings (Croke, 2017; de Kadt & Lieberman, 2017; Fried and Atheendar, 2017).

Political trust is an assessment by citizens that their government will act in ways that are consistent with citizen well-being in the absence of monitoring (Miller and Ola, 1990). When citizens trust the government, they are more likely to comply with rules and regulations, reducing the cost of enforcement and governance (Marien & Hooghe, 2011; Murphy, 2004). Voluntary compliance is particularly important when the costs of adhering to rules and regulations exceed the individual returns to compliance, where the mechanisms for enforcement are expensive, or where short-term benefits need to be sacrificed for longer-term gains (OECD, 2015). Thus, a high level of trust in government promotes healthy state-society relations, particularly in environments where regulatory enforcement is weak, as in several African countries (Aberbach & Walker, 1970; Evans, Holtemeyer, & Kosec, 2019).

Nigeria provides an interesting context in which to study the linkages between service delivery and political trust, with characteristics which are different from the Western countries that are the focus of most of the existing empirical literature on political trust. A central feature of the Nigerian political economy that has implications for patterns of service delivery is the presence of religious and ethnically-defined blocs, which compete for direct representation in government to control state resources (Joseph, 1987, 2018). This competition is characterized by the willingness of groups to sacrifice material benefits for advantages that improve the group's status and place in the larger society, and each group considers their interests threatened by the advancement of other groups (Horowitz, 2001). .

Because of these dynamics, we hypothesize that group identity may be an important mediator of the impact of improvements in health service delivery on political trust in Nigeria (Carlson, 2015; Conroy-Krutz and Jeffrey, 2013). If changes in political trust reflect the objective evaluation of the performance of political actors and organizations, then improvements in service delivery should lead to higher levels of political trust. However, if variation in trust in political leaders simply reflects beliefs that already exist about ethnic and religious groups in a society, then these beliefs will constrain evaluations of service delivery improvements (Mishler & Rose, 2001). There is an expansive body of empirical studies, predominantly from Western countries, which show that political trust is determined both by the performance of political institutions and by pre-existing beliefs about the identity of political actors and institutions (Andersen & Tverdova, 2003; Brinkerhoff, Wetterberg, and Wibbels 2018; Catterberg, 2006; Christensen & Lægreid, 2005; Godefroidt, Langer, & Meuleman, 2017; Lavallée, Emmanuelle; Razafindrakoto, Mireille; Roubaud 2008; Levi & Stoker, 2000; Shi, 2001; Zhao & Hu, 2017). The literature examining the health-related determinants of political trust in African countries is less expansive and does not explore a mediating role for group identity (Rockers and Miriam, 2012). Thus, in this paper, we examine if there is a role for ethnic and religious identity in mediating the impact of health service delivery on political trust in the Nigerian president.

Our focus on health service delivery in this paper is intentional. In the most recent round of Afrobarometer surveys across 36 countries in Africa, citizens ranked health as the second most important problem, after unemployment, that required additional government investments (Armah-Attoh, Selormey, & Houessou, 2016). In comparison to other regions of the world, Sub-Saharan Africa has low ratings for wellbeing and the lowest satisfaction with health service delivery (Deaton and Robert, 2015). Access to quality health care is vital to good health. Furthermore, services delivered through the health care system were identified as an essential social determinant of population health by the World Health Organization's Commission on Social Determinants of Health (Commission on Social Determinants of Health, 2008). However, assessments of access to quality health care place most African countries, including Nigeria, in the lowest two deciles globally (Fullman et al., 2018). In 2015, government health spending in African countries averaged seven percent of general government expenditure, far below the target of 15 percent that the African Union committed to through the Abuja Declaration (WHO, 2019). The Millennium Development Goals and the 2030 Agenda for Sustainable Development have included commitments by African Governments to expand access to health services to improve population health outcomes (UN-MDG, 2015; United Nations General Assembly, 2015). While politicians may have intrinsic interest in improving the health of their citizens, they also care about maintaining political support. Thus in evaluating the impact of improvements in health service delivery on outcomes related to their support levels, such as political trust, we can examine the incentives that politicians face as they make decisions about the service improvements necessary to expand access to quality healthcare under the 2030 Agenda and improve population health.

In this paper, we estimate the impact of a national intervention that improved health service delivery in 500 primary health care facilities in Nigeria on trust in political leaders and institutions. We spatially matched the geocoordinates of facilities selected for the intervention with clusters of respondents in a nationally-representative survey of political attitudes and behavior. We examined the impact of residence within 10 km of one or more of the intervention facilities on trust in the president, local council, ruling party, and official parties. We show that proximity to the intervention facilities led to increases in trust in the president and ruling party. We find no comparable evidence of increases in trust in the local council, and opposition parties. Our study also indicates that there may be a role for group identity, particularly ethnicity and religious affiliation, in mediating the increases in trust in the president following the intervention. The intervention's effect was larger for individuals who did not live in the president's home state and zone, and who were not of his ethnicity. This suggests that political trust also increased in response to positive evaluations of healthcare investments that were partially mediated by group identity.

The rest of this paper is organized as follows: Section 2 describes the relevant political and health sector context under study; Section 3 describes the study dataset and the identification strategy; Section 4 discusses the results, and Section 5 concludes.

## 2 Context

There are three tiers of Government enshrined in the Nigerian Constitution: the federal government, 36 semi-autonomous states and the federal capital territory (FCT), and 774 local government areas. The president, who heads the executive branch of the Federal Government, is elected every four years by universal adult suffrage. Major political parties rotate the states from which the president is nominated in pursuance of equitable sub-national access to the state's resources. Since the transition from military to democratic rule in 1999, Nigeria has held presidential elections in 2003, 2007, 2011, and 2015. The candidate of the People's Democratic Party (PDP) won the presidential elections in each year but 2015, when the All Progressives Congress (APC) Party won the election. This transition in power was the first time in Nigeria's political history that an opposition political party won the presidency. State governors are elected every four years and elected executive chairpersons administer LGAs.

Primary care is the lowest level of preventative and curative health services in Nigeria, provided through health posts, health clinics, health centers, and comprehensive health centers (Uzochukwu, 2017). The health departments of LGAs are primarily responsible for coordinating PHC operations. However, allocations decided at the federal level flow through a range of actors to PHC facilities. Therefore, the executive arms of the federal and local government significantly influence the financing and management of PHC in Nigeria. There are 34173 health facilities listed in the National Health Facility Directory in Nigeria, of which 88 percent are PHC facilities and 66 percent are government-owned (Uzochukwu, 2017). While there is some variation, on average PHC facilities in Nigeria have a low capacity for service provision (NPHCDA, 2015). Only a quarter of PHCs have up to 25 percent of the minimum required equipment, and only 20 percent provide basic emergency obstetric services. The poor quality of health services available through PHC in part explains the bypassing of these facilities for more expensive and less accessible secondary and tertiary-level care (NPHCDA, 2015; Uzochukwu, 2017).

**Fig. 1:**
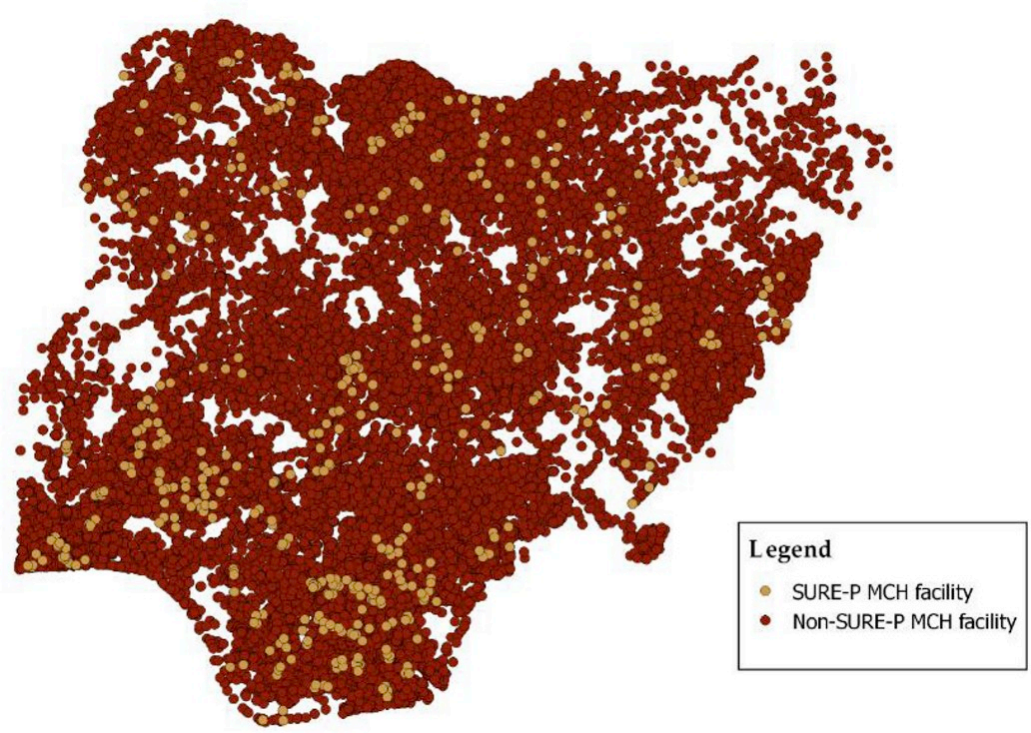
Distribution of SURE-P MCH facilities.

On January 1, 2012, the federal government announced the discontinuation of a state subsidy programme which had kept fuel cheap for decades, doubling the petrol pump price. As a result, there were protests in the federal capital territory and states across the country, involving sit-ins in major urban centers (Monica Mark, 2012). In response to the gaps in quality of care and to quell the national protests, the Nigerian President launched a national health program, the Nigerian Subsidy Reinvestment and Empowerment Programme Maternal and Child Health Intervention (SURE-P MCH), which was funded using savings from the removal of fuel subsidies. The investments in facility upgrading and staffing started in January 2012 and were completed by October 2012. Each state and the Federal Capital Territory selected nine to sixteen PHC facilities for the intervention. SURE-P MCH was implemented in a total of 500 PHC facilities or 1.7 percent of all PHCs in the country. The criteria for selecting these facilities were: location in a rural area; a catchment population of more than 10,000 residents; offering maternal and child health services; availability of minimum equipment and basic infrastructure including potable water supply, power supply, and sewage disposal; and operating twenty-four hours daily (Fig. 1). These criteria were selected by the National Primary Health Care Development Agency to reflect the service delivery needs of the population and the capacity of the facilities for care.

The National Primary Health Care Development Agency oversaw improvements in service delivery in the selected facilities. The SURE-P MCH intervention included:

1. Deploying unemployed, retired, and newly-graduated midwives to provide skilled care within health facilities;
2. Deploying trained community health extension workers and village workers for health promotion in the facility catchment areas;
3. Refurbishing the health facility buildings; ensuring availability of essential medical supplies for maternal and child health care;
4. Providing financial and non-financial incentives to encourage retention of midwives in the health facilities; and
5. Conditional cash transfers for attendance of antenatal, delivery, and postnatal care among mothers.

There was also a nationwide mass media campaign, not limited to the catchment areas of SURE-P MCH facilities, encouraging visits to PHC facilities for health care. The mass media campaign involved jingles on radio and television, billboards and posters that attributed the intervention to the Presidency.

The SURE-P program represented a significant national commitment by the FMOH. In a 2013–2014 fiscal space analysis for health in Nigeria, the allocation to SURE-P MCH was USD 105.7 Million, equivalent to 6 percent of the total budgetary allocation to health of USD 1, 848.3 Million (R4D, 2014). From 2015 to date, financial support for the interventions started under the SURE-P program has been continued through the USD 500 Million Saving One Million Lives Initiative, supported by the World Bank. A 2014 progress report noted that SURE-P MCH had deployed 11896 health workers, undertaken 74 facility renovations, and constructed 313 boreholes for water supply. By improving the supply of essential drugs and health commodities, informal user fees for maternal and child care were noted to have been reduced. Advocacy visits, led by the National Primary Health Care Development Agency, were also conducted in communities within the catchment areas of intervention facilities and committees were instituted to monitor health service delivery (CSJ, 2014). Interviews with program staff indicate that the SURE-P MCH intervention was well received in recipient communities, including those communities that received only supply-side interventions (UCSF Global Health Group, 2014).

The SURE-P MCH intervention also led to increases in maternal health care use. A non-experimental impact evaluation of SURE-P MCH indicates that mothers who lived in catchment areas of intervention facilities were 12 percentage points more likely to receive skilled support from a doctor, nurse, or midwife at child birth than they would have been without the project (Holmlund & Crawford, 2016). This effect is notable as the prevalence of skilled birth attendance had remained unchanged in the decade preceding the SURE-P MCH intervention (National Population Commission (NPC) [Nigeria] and ICF Macro 2009; National Population Commission of Nigeria, 2014). Skilled support during childbirth can prevent maternal and neonatal mortality, making the impact of the SURE-P MCH intervention on health care use significant for population health in Nigeria (Bhutta et al., 2010). The rest of this paper evaluates the impact of the SURE-P MCH intervention on political trust.

## 3 Dataset and identification strategy

We used the Afrobarometer surveys, which are nationally representative cross-sectional datasets, involving face-to-face interviews of citizens of voting age in Africa. Afrobarometer has conducted surveys on public attitudes and opinions on a range of issues including democracy, governance, and economic conditions, in 36 countries. In Nigeria, six rounds of the Afrobarometer survey have been conducted in 1999, 2003, 2005, 2008, 2012, and 2015. We restricted the study sample to rounds 2003–2015, over which the variables we intended to explore were collected. Fieldwork for the 2012 survey round was conducted in October–November 2012, while field work for the 2015 survey round was conducted between December 2014 and January 2015. Therefore, the survey rounds two to four (2003–2008) were conducted pre-intervention. Survey rounds five and six (2012–2015) occurred post-intervention.

Each survey uses a multistage, clustered, stratified probability sampling design. First, the voting population is stratified by urban and rural location. Secondary sampling units (SSUs) are identified, in which were nested primary sampling units (PSUs) or clusters. A random sample of SSUs is then selected. In rural areas, two clusters are randomly sampled from each SSU. In urban areas, clusters are sampled with probability proportional to the population size. Within each cluster, enumerators begin at a randomly-selected starting point and sample eight households for interviews in a systematic walk pattern. The gender of respondents is alternated for each interview. The sample size for each survey is about 2400 cases, yielding a margin of sampling error of plus or minus 2·0 percentage points at the 95% confidence interval. The Afrobarometer dataset includes geocoordinates of the clusters enabling us to spatially match these locations with the facilities selected for the SURE-P MCH intervention (BenYishay, Rotberg, et al., 2017a,b).

The catchment area is an intuitive way to define physical proximity to health facilities. The facility catchment area is the maximum distance between an individual's place of residence and the nearest health facility to ensure health care access. There is no official definition of a facility catchment area in Nigeria. The distances used also vary across similar contexts in Sub-Saharan Africa, from 5 km in Zambia and 8 km in Malawi to 10 km in Mali (Guenther et al., 2012). There is thus no consensus on the appropriate distance to consider in determining physical access to health facilities when there is no policy target. Narrowing the target distance used to define the SURE-P MCH facility catchment area would progressively include individuals with access to equivalent or better non-SURE-P MCH care in the comparison group. Thus, we use the highest facility catchment area distance (10 km) to construct the exposure variable, and we examine the impact of variation in this distance on the studied outcomes as a robustness check. Furthermore, by matching the clusters of respondents in the sample to geocoordinates in the National Health Facility Directory, we determined that 99 percent of the sample lives within 10 km of any health facility in each of the included survey rounds.

**Fig. 2:**
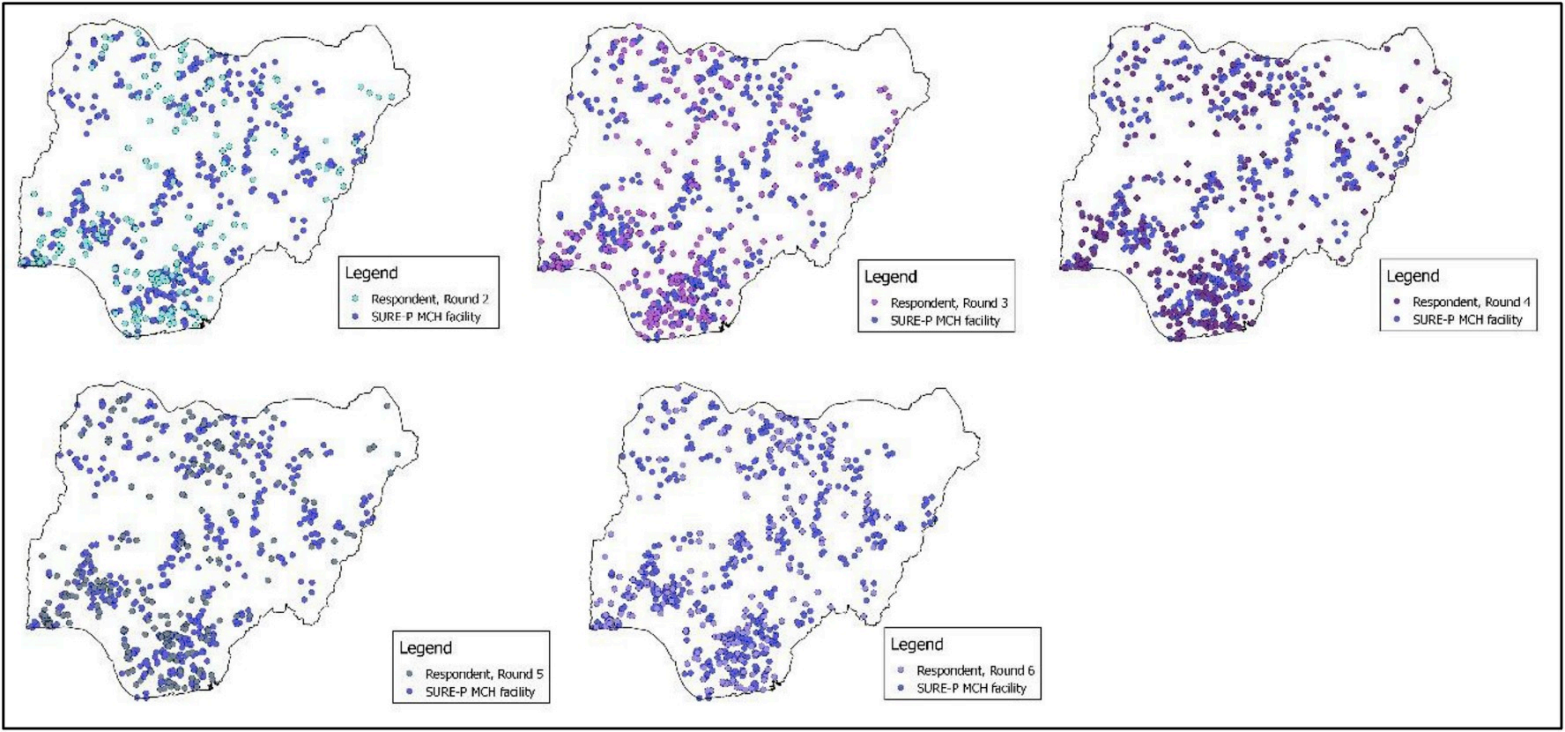
SURE-P MCH facility distribution relative to survey respondent locations.

To construct a measure of proximity to SURE-P MCH facilities, we determined the distance between the geocoordinates of each survey cluster and all the facilities selected for the SURE-P MCH intervention (SURE-P MCH facilities). Of the 500 facilities, geocoordinates are available for 459 SURE-P MCH facilities. The distribution of the facilities selected for the SURE-P MCH intervention relative to respondent clusters in each survey round is shown in Fig. 2above. In Table 1, we show that the number of SURE-P MCH facilities within 10 km of a respondent's cluster varied from 0 (69.79 percent of the sample across surveys) to 6 (2.25 percent of the sample across surveys). The binary intervention variable in this study classified respondents as follows: no SURE-P MCH facility within 10 km of the respondent's cluster (0) and, one or more SURE-P MCH facility or facilities within 10 km of the respondent's cluster (1).

**Table 1:** Number of SURE-P MCH facilities within 10 km of respondents.

#	2003 N (%)	2005 N (%)	2008 N (%)	2012 N (%)	2015 N (%)	Total
0	1672 (68.86)	1602 (67.80)	1634 (70.31)	1728 (72.00)	1680 (70.00)	8316 (69.79)
1	267 (11.00)	301 (12.74)	286 (12.31)	304 (12.67)	296 (12.33)	1454 (12.20)
2	138 (5.68)	125 (5.29)	86 (3.70)	80 (3.33)	168 (7.00)	597 (5.01)
3	184 (7.58)	161 (6.81)	142 (6.11)	144 (6.00)	88 (3.67)	719 (6.03)
4	31 (1.28)	80 (3.39)	112 (4.82)	48 (2.00)	88 (3.67)	359 (3.01)
5	50 (2.06)	32 (1.35)	40 (1.72)	32 (1.33)	48 (2.00)	202 (1.70)
6	86 (3.54)	62 (2.62)	24 (1.03)	64 (2.67)	32 (1.33)	268 (2.25)
**Total**	**2428 (100)**	**2363 (100)**	**2324 (100)**	**2400 (100)**	**2400 (100)**	**11915 (100)**

Notes: N refers to the number of respondents; # refers to the number of SURE-P MCH facilities within 10 km of respondents.

As discussed above, the executive arms of the federal and local government largely influence the financing and management of PHC in Nigeria. Of the three levels of the government, the data on trust is collected consistently across survey rounds for the executive in the federal and local levels: the president and the local executive council. Given the role that political parties play in the selection of presidential candidates, we also study trust in the ruling and opposition party. This data is collected consistently across survey rounds as well. The surveys measured trust in these individuals or organizations on a 4-point scale. Respondents indicated if they trusted the President/local council/ruling party/opposition parties not at all (0), a little bit (1), a lot (2), or a great deal (3). For each outcome, we derived a binary variable whose value was (1) if the respondent indicated that they trusted the individual or organization a lot or a great deal and (0) otherwise. These were the dependent variables for this study.

In addition to the intervention, our main models included explanatory variables that adjusted for demographic factors and selection criteria for SURE-P MCH locations. These variables include: residence in an urban area, age in years, attaining up to secondary school education, and employment status. The President who launched the SURE-P MCH intervention identified as a Christian from the Ijaw tribe, from the “South-South” geopolitical zone, and from Bayelsa State. He ran on the platform of the PDP. While the Afrobarometer does not allow us to clarify the party, religious and ethnic identity of local leaders, these variables are more homogenous within local government areas. To explore the influence of group identity on the relationship between the intervention and political trust, we included the following binary variables: identification as a Christian, identification as a member of the Ijaw tribe, residence in the South-South geopolitical zone, and residence in Bayelsa state.

We modeled political trust as a function of the intervention variable, and demographic factors, using difference-in-difference models. For each measure of trust, we specified a linear probability model as follows:

$$Y_{ijt}=\alpha_{ij}+\;\gamma *SUREP_{ijt}+\beta Post_{t}+\;\delta \left(SUREP_{ijt}*Post_{t}\right)+X_{ijt}+\varepsilon_{ijt}$$

where the level of trust expressed in the politician or political institution is $\left(Y_{ijt}\right)$ for respondent *i* in PSU *j* at time *t*; $X_{ijt}$ is a vector of socioeconomic and demographic time-varying covariates (age, education, urban/rural residence, and employment status); $SUREP_{ijt}$ is equal (0) if there is no SURE-P MCH facility within 10 km of the respondent's cluster, and (1) if there is one or more SURE-P MCH facility or facilities within 10 km of the respondent's cluster; $Post_{t}$ is equal to 1 if the respondent was surveyed in the 2012 or 2015 rounds and 0 otherwise; δ is the DID estimate if the identifying assumptions discussed briefly below hold; and the standard errors are robust and clustered at the PSU level. Sampling weights were applied in the main model and sensitivity of findings to these weights were examined as robustness checks. The empirical model estimates the impact of the intervention on political trust under two assumptions. First, that in the absence of the intervention, the difference between levels of political trust would remain constant over time, that is trends in the levels of political trust would be parallel. We present evidence suggesting that we cannot reject the null hypothesis of parallel trends as a robustness check. Another key assumption is that within the study period, there was no other national intervention with a distribution akin to the nationwide SURE-P MCH intervention took place, which is accurate to our knowledge.

## 4 Results

We first characterize the clusters which were closer to the SURE-P MCH intervention facilities, by examining the baseline differences in respondent characteristics as described in Table 2 below. We see that at baseline, respondents in intervention clusters were less likely to express trust in the President, local council, ruling party, or opposition parties. Regarding group identity, respondents in intervention clusters were more likely to be Christian. However, there was no difference in the proportion of respondents in intervention versus control clusters that identified as Ijaw, who resided in Bayelsa state, or who were from the South-South geopolitical zone. Intervention clusters were more likely to have pipe borne water and electricity. There was no significant difference in the probability of having a clinic or a sewage system between intervention and control clusters. Intervention clusters were more likely to be urban even though a rural location was a criterion in facility selection for SURE-P MCH. Respondents in intervention clusters were less likely to consider health care a priority, approve of health service delivery, or to have had to forgo medical care in the past one year. Respondents in intervention clusters were also more likely to be educated and employed.

**Table 2:** Pre-intervention differences between intervention and control communities.

Variable	Control	Intervention	Intervention - Control	95% CI
Trusts the President a lot/a great deal	0.335	0.203	−0.131	−0.166, −0.096
Trusts the local council a lot/a great deal	0.257	0.162	−0.095	−0.125, −0.064
Trusts the ruling party a lot/a great deal	0.259	0.149	−0.110	−0.142, −0.078
Trusts opposition parties a lot/a great deal	0.257	0.168	−0.089	−0.119, −0.058
Age (years)	31.950	31.064	−0.885	−1.655, −0.116
Up to secondary school education	0.606	0.789	0.182	0.142, 0.222
Urban residence	0.399	0.676	0.277	0.205, 0.349
Employed	0.449	0.500	0.051	0.017,0.084
Clinic located in cluster	0.652	0.658	−0.006	−0.065, 0.077
Pipe-borne water in cluster	0.367	0.470	0.102	0.028, 0.177
Electrical connections in cluster	0.726	0.871	0.145	0.087, 0.203
Sewage system in cluster	0.265	0.328	0.063	−0.006, 0.133
Identification as Ijaw	0.045	0.043	−0.002	−0.022, 0.020
Identification as Christian	0.480	0.696	0.215	0.158, 0.272
Lives in Bayelsa state	0.038	0.048	0.010	−0.016, 0.037
Lives in South-South zone	0.148	0.160	0.012	-.036, 0.060
Forgone medical care in household over past one year	0.619	0.522	−0.097	−0.137, −0.056
Considers health or health care a priority issue	0.179	0.115	−0.064	−0.087, −0.040
Approves of basic health service delivery	0.477	0.410	−0.067	−0.103, −0.031
**N**	**4908**	**2207**	**7115**	

Notes: The table shows means across intervention and control respondents for each dependent and independent variable in the study. Coef. Refers to the coefficient of a simple regression of the listed variable as outcome and intervention assignment status as the independent variable, with standard errors clustered at the level of the sampling unit.

Taken together, these characteristics provide descriptive evidence against arguments that SURE-P MCH locations were chosen exclusively on the objective need for health care. SURE-P MCH facilities were located closer to respondents with relatively higher socioeconomic status, who were less likely to consider health service improvements a priority, and who were less likely to express trust in or approve of the Government. However, there does not appear to have been any direct targeting of the President's Ijaw ethnic group, Bayelsa state, or South-South geopolitical zones. The preconditions for facility inclusion in the SURE-P MCH program – electricity, piped water, and sewage disposal systems –may have led to a pattern of higher intervention exposure among educated and employed urban dwellers.

In Table 3, we present our main results: any exposure to the SURE-P MCH intervention predicts increases in trust in the President and ruling party. We cannot reject the null hypothesis that there was no increase in trust in the local council and opposition party because of the intervention. From a low average of 30.1 percent and 25.7 percent of respondents located in clusters with no SURE-P MCH facility within 10 km at baseline, the intervention increased trust in the President and ruling party by 10.2 percentage points and 7.6 percentage points, respectively, if any SURE-P MCH facility was located within this distance.

**Table 3:** Intervention effect on political trust.

Variable	Trusts politician or institution a lot or a great deal			
	President	Local council	Ruling party	Opposition parties
SUREP*Post [95% CI]	0.102 [0.045, 0.159]	0.024 [-0.027, 0.075]	0.076 [0.025, 0.127]	0.019 [-0.029, 0.068]
SUREP [95% CI]	−0.124 [-0.160, −0.089]	−0.070 [-0.100, −0.040]	−0.095 [-0.127, −0.063]	−0.071 [-0.102, −0.039]
Post [95% CI]	−0.002 [-0.036, 0.033]	0.038 [0.009, 0.068]	0.035 [0.004, 0.066]	0.041 [0.011, 0.071]

Control [95% CI]	0.301 [0.258, 0.345]	0.316 [0.277, 0.355]	0.257 [0.218, 0.296]	0.318 [0.281, 0.356]
F-stat	12.320	18.400	13.680	13.050
**N**	**11839**	**11839**	**11839**	**11839**

Notes: All empirical models adjust for the following covariates: residence in an urban area, age in years, attaining up to secondary school education, and employment status. Standard errors are clustered at the level of the Afrobarometer cluster and robust. Sample weights are applied.

In Table 4, we present evidence in support of the robustness of our main findings. We examined the sensitivity of our findings to the non-inclusion of sampling weights or socioeconomic and demographic covariates, and to restriction of the control sample to respondents located in clusters for which the nearest facility was within 10 km. In each case, there are increases in trust in the President and ruling party in clusters that received the SURE-P MCH intervention and the size of the coefficients is comparable to those in the main models. As in the main models, we cannot reject the null hypotheses in the case of local council and opposition party in these models (see Table 5).

**Table 4:** Robustness checks.

	Variable	Full model, no sampling weights	No controls	Limited to respondents with any facility within 10 km	State fixed effects
Trusts president a lot or a great deal	SUREP∗Post [95% CI]	0.103 [0.049, 0.158]	0.103 [0.046, 0.159]	0.103 [0.046, 0.160]	0.082 [0.025, 0.139]
Trusts local council a lot or a great deal	SUREP∗Post [95% CI]	0.021 [-0.027, 0.069]	0.026 [-0.025, 0.078]	0.025 [-0.025, 0.077]	0.018 [-0.030, 0 .066]
Trusts ruling party a lot or a great deal	SUREP∗Post [95% CI]	0.071 [0.023, 0.119]	0.075 [0.023, 0.127]	0.077 [0.026, 0.129]	0.058 [0.011, 0.106]
Trusts opposition parties a lot or a great deal	SUREP∗Post [95% CI]	0.029 [-0.017, 0.075]	0.021 [-0.028, 0.070]	0.019 [-0.029, 0.068]	0.002 [-0.0426339, 0 .0475436]
	**N**	**11839**	**11839**	**11839**	**11839**

**Fig. 3:**
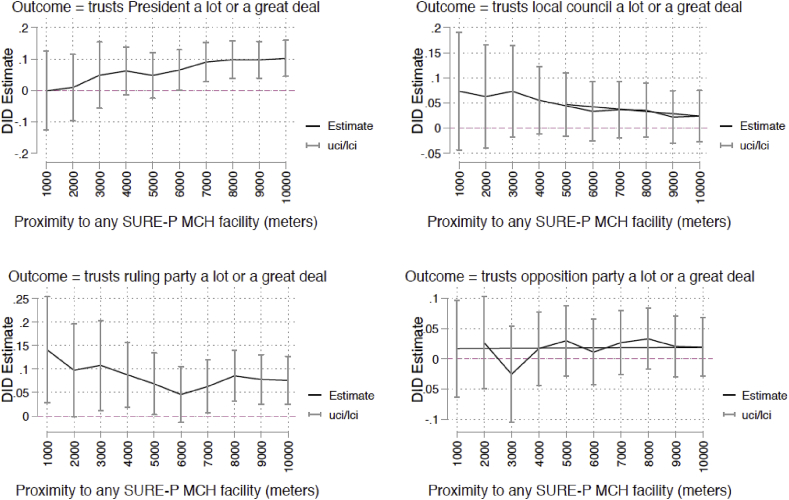
Variation in facility catchment area definition.

We also examine the robustness of our findings to variation in the distance used to define the facility catchment area. We vary the distance in 1-km increments from 1 km to 10 km and re-estimate the main models for each of the dependent variables. These distances represent the range of values used to define national targets for proximity to health care facilities in the population in Africa. We cannot reject the null hypotheses that there is no impact of the SURE-P MCH intervention on trust in the local council and opposition party across the range of distances. We find that the SURE-P MCH intervention leads to increases in trust in the President when the distance to the nearest intervention facility is 6 km–10 km. The intervention also predicts increases in trust in the ruling party between 1 km and 10 km (Fig. 3).

**Fig. 4:**
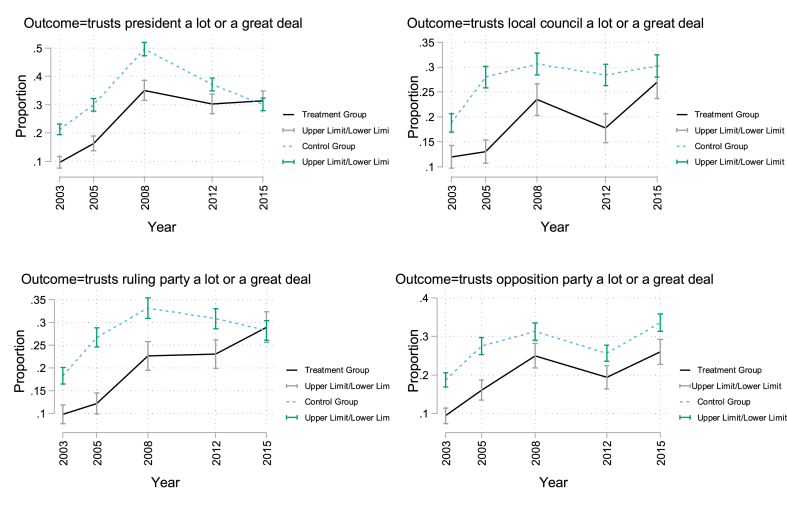
Pre- and post-intervention trends in outcome.

The key identifying assumption in our empirical model is that in the absence of the intervention, the mean differences in political trust between intervention and control clusters would have been relatively constant. We present evidence below in support of this intervention for the outcome measuring trust in the president. First, we plot graphs of the mean levels of trust before and after the intervention. We then test this assumption formally by re-estimating the main models using data limited to the three survey rounds in the pre-intervention period. The graphs suggest that in the pre-intervention period, trends in mean values are reasonably parallel across levels of the intervention for trust in the President, trust in the ruling party, and trust in the opposition party (Fig. 4). For trust in the local council, there is significant deviation from parallel trends in the pre-intervention period, suggesting that the identifying assumption does not hold. To test the assumption of parallel trends prior to the intervention, we generate a placebo treatment taking place in the period preceding the actual treatment, assuming the intervention began after 2005, with 2008 as the placebo post-intervention survey round. We restricted the sample to the three survey rounds [2003, 2005, and 2008] before the intervention. We could not reject the null hypothesis that the placebo treatment coefficients were different from zero across study outcomes (Table 5).

**Table 5:** Parallel trends analysis.

Variable	Trusts politician or institution a lot or a great deal			
	President	Local council	Ruling party	Opposition parties
SUREP*Post [95% CI]	−0.023 [-0.093, 0.047]	0.034 [-0.027, 0.097]	0.006 [-0.065, 0.077]	0.037 [-0.031, 0.104]
SUREP [95% CI]	−0.109 [-0.143, −0.075]	−0.079 [-0.113, −0.044]	−0.096 [-0.129, −0.062]	−0.088 [-0.121, −0.055]
Post [95% CI]	0.245 [0.201, 0.290]	0.077 [0.036, 0.118]	0.110 [0.068, 0.151]	0.089 [0.050, 0.127]
Control [95% CI]	0.259 [0.211, 0.306]	0.307 [0.260, 0.354]	0.258 [0.215, 0.302]	0.292 [0.246, 0.339]
F-stat	49.620	18.590	18.660	16.770
**N**	**7115**	**7115**	**7115**	**7115**

Notes: All empirical models adjust for the following covariates: residence in an urban area, age in years, attaining up to secondary school education, and employment status. Standard errors are clustered and robust. Sample weights are applied.

These robustness checks strengthen our confidence that the SURE-P MCH intervention caused an increase in trust in the President and the ruling party in intervention clusters relative to control clusters. These findings are plausible considering the nationwide mass media campaign which highlighted that the SURE-P MCH intervention was an initiative of the Presidency. Attribution of service delivery improvements to the President and the ruling party, rather than the local council or opposition parties, could be thus expected.

We also explore the role of group identity in mediating the effect of SURE-P MCH on trust in the President. In Table 6, we compare the effect of SURE-P on trust for respondents who share some marker of identity with the president (ethnicity, geopolitical zone, state of residence, and religion) with those who do not share these identities, using triple difference models. We present evidence suggesting that there were higher increases in trust in response to the intervention among those who do not share certain identity markers with the president, such as geopolitical zones or state of residence. The differences are large: for example, the treatment effect outside the South-South zone is 13 percentage points; by contrast, for residents of South-South zone, the effect is −0.01. The interaction terms for the triple-difference coefficients for Bayelsa state residence and South-south geopolitical zone are marginally significant at the 0.1 level, with p-values of 0.09. By contrast, religion does not appear to drive differences in the variation in trust in the president in response to the interventions, with a p-value on the coefficient of 0.47.

**Table 6:** Group identity and trust in the President.

Variable	Ijaw	Christian	South-South zone	Bayelsa state
SUREP*Post*Variable [95% CI]	−0.260 [-0.529, 0.008]	−0.037 [-0.138, 0.060]	−0.139 [-0.298, 0.021]	−0.228 [-0.490, 0.034]
SUREP*Post [95% CI]	0.115 [0.057, 0.173]	0.083 [0.004, 0.162]	0.129 [0.068, 0.190]	0.111 [0.053, 0.169]
SUREP [95% CI]	−0.130 [-0.168, −0.094]	−0.123 [-0.182, −0.063]	−0.138 [-0.178, −0.098]	−0.129 [-0.166, −0.093]
Post [95% CI]	−0.017 [-0.052, 0.018]	−0.139 [-0.036, 0.034]	−0.052 [-0.089, −0.015]	−0.015 [-0.050, 0.020]

Constant [95% CI]	0.307 [0.265, 0.351]	0.363 [0.317, 0.409]	0.325 [0.281, 0.368]	0.309 [0.266, 0.352]
F-stat	712.130	18.880	14.000	16.180
**N**	**11839**	**11839**	**11839**	**11839**

Notes: All empirical models adjust for the following covariates: residence in an urban area, age in years, attaining up to secondary school education, and employment status. Standard errors are clustered and robust. Sample weights are applied.

## 5 Conclusion

This paper adds to a growing body of research that causally estimates the links between health service delivery and political support in Sub-Saharan Africa. We show that proximity to improved maternal and child health services led to increases in trust in the President and ruling party, but not in the local council and opposition parties. These findings suggest that efforts to ensure attribution of the SURE-P MCH intervention to the Presidency via a nationwide mass media campaign were successful.

Our study indicates that there was a role for ethnicity and religious affiliation in mediating the increases in trust in the President following the intervention. These findings are in some tension with theories that predict that beliefs based on group identity may constrain assessments of performance in some contexts. The suggestive evidence of a positive effect of the intervention on non-members of the President's ethnic group suggests that even in settings where ethnic and religious affiliation influence political support, there may also be variation in trust in the President in response to improved services. This finding that citizens may update their evaluation of politicians in response to health programs, is consistent with evaluations of political returns to health investments in Mexico and Tanzania (Croke, 2017; Fried and Atheendar, 2017). However, in South Africa, improvements in service provision predicted decreases in support for the ruling party, potentially due to concerns about corruption (de Kadt & Lieberman, 2017). The differences in impact of service delivery improvements across these settings are likely explained by context-specific mechanisms. Thus, the roles of group identity in Nigeria and corruption in South Africa in mediating the responses of citizens to improvements in health services should inform discussions on the generalizability of findings to different contexts.

A comparison of intervention and control communities indicates that the location of SURE-P MCH facilities may not have coincided with a higher need for improved health services. Citizens who lived closer to intervention facilities were more likely to be educated, employed, and urban. These characteristics predict a higher probability of using skilled care at delivery at baseline. For example, while 11.7 percent of women with no education use skilled care at delivery, 71.7 percent of women with secondary education and 93.2 percent of women with more than secondary education use skilled care at delivery in Nigeria (National Population Commission of Nigeria, 2014). Residents of the intervention areas were also less likely to support local and national politicians at baseline, raising questions about potential political considerations in selecting facilities for SURE-P MCH. However, the selection of facilities may also have reflected the minimum criteria for service delivery such as availability of power and water supply, commonly found in wealthier communities. These criteria were contradictory in some cases. For example, power and water supply are more likely to be found in urban than rural locations. This may explain why intervention communities were more likely to be urban than control communities, despite the selection criteria.

Integrating spatial data on facility location and a retrospectively-georeferenced nationally-representative survey of political attitudes enabled us to examine this research question, despite the non-random assignment of the SURE-P MCH intervention. However these datasets do have limitations. First, we are unable to explore interesting lines of inquiry due to data limitations, including sub-group analyses focusing on the pregnant women targeted by the SURE-P MCH intervention, and differentiating intervention and control communities according to the priority given to maternal and child health services specifically, rather than all basic health services. Secondly, the geo-referenced locations of 41 out of 500 facilities selected for phase one of the SURE-P MCH intervention were missing from the study dataset, potentially leading to an underestimate of the impact of the intervention on political trust. Despite the limitations of these methods, with the increased availability of national and subnational program and outcome data with similar spatial and temporal scales, geospatial impact evaluations provide an alternative method to evaluate interventions in situations when field experimentation is infeasible or unethical (BenYishay, Rotberg, et al., 2017a,b).

Politicians may have intrinsic interest in improving the health of their citizens. In evaluating the impact of improvements in health service delivery on trust, we aimed to examine the political incentives that African politicians face as they undertake the service improvements necessary to expand access to quality healthcare. As in Nigeria, ethnic and religious affiliation have been shown to influence political support in several other countries in Africa. Our findings suggest that in contexts where ethnic and religious identity influence politics, the benefits that leaders enjoy due to improved health service delivery are not necessarily limited to members of their own group. The body of literature exploring the links between political support and health care investment calls to attention the importance of political considerations, in addition to health-specific objectives, in shaping the choices that governments make in implementing their commitments towards Universal Health Coverage.

## Financial disclosure statement

None.

## Ethics, consent and permissions

This study was a secondary analysis of anonymous data from the Afrobarometer Surveys, collected as part of research protocols with ethical approvals obtained at the University of Capetown. Formal approval to use the data was obtained from the Afrobarometer program. The team obtained permission to use the SURE-P MCH program data from the principal investigators: Marcus Holmlund (Economist, World Bank Group), Marcos Vera-Hernández (Reader, University College, London), and Pedro Rosa Dias (Associate Professor, Imperial College London).

## Conflicts of interest

The motivations, findings, interpretations, and conclusions expressed in this work do not necessarily reflect the views of The World Bank, its Board of Executive Directors, or the governments they represent. The World Bank does not guarantee the accuracy of the data included in this work. The boundaries, colors, denominations, and other information shown on any map in this work do not imply any judgment on the part of The World Bank concerning the legal status of any territory or the endorsement or acceptance of such boundaries. The authors declare that they have no competing interests.
